# Comparative Efficacy of Skin-Lightening Formulations in Suppressing Ultraviolet B (UVB)-Induced Arachidonic Acid, Alpha-Melanocyte-Stimulating Hormone (α-MSH), and Melanin Expression: An In Vitro Keratinocyte-Melanocyte Co-culture Study

**DOI:** 10.7759/cureus.78908

**Published:** 2025-02-12

**Authors:** Simone Ribero, Andrea Romani, Carlo Mattozzi, Piercarlo Minoretti

**Affiliations:** 1 Medical Sciences, University of Turin, Turin, ITA; 2 Dermatology, Outpatient Clinic Romani, Montecatini Terme, ITA; 3 Dermatology, Outpatient Clinic Mattozzi, Marsala, ITA; 4 Occupational Health, Studio Minoretti, Oggiono, ITA

**Keywords:** alpha-melanocyte-stimulating hormone, arachidonic acid, keratinocyte-melanocyte co-culture model, melasma, skin-lightening formulations, ultraviolet radiation

## Abstract

Background: Melasma is a chronic hyperpigmentary disorder exacerbated by sun exposure, characterized by asymptomatic hyperpigmented macules and patches. Its pathogenesis involves ultraviolet B (UVB)-induced activation of melanogenesis pathways, including arachidonic acid (AA) metabolism, alpha-melanocyte-stimulating hormone (α-MSH) secretion, and melanin synthesis. This study aimed to evaluate the efficacy of a multi-ingredient skin-lightening formulation (3% tranexamic acid, 2% ascorbyl glucoside, 2.5% arbutin, and 5% niacinamide) compared to a 10% vitamin C preparation and 4% hydroquinone (HQ) in suppressing UVB-induced AA, α-MSH, and melanin production in a keratinocyte-melanocyte co-culture model.

Methods: Keratinocyte-melanocyte co-cultures were pretreated with the multi-ingredient formulation, 10% vitamin C, or 4% HQ before UVB irradiation (150 mJ/cm^2^). Distilled water served as the negative control. Biomarkers (AA, α-MSH, and melanin) were quantified using enzyme-linked immunosorbent assay (ELISA) and fluorometric assay.

Results: UVB irradiation significantly increased AA (4.3-fold), α-MSH (3.7-fold), and melanin (2.6-fold) levels compared to unirradiated cells (all p < 0.001). All tested formulations reduced AA levels compared to the negative control (p < 0.001), with the multi-ingredient formulation demonstrating superior efficacy over both 10% vitamin C and 4% HQ (p < 0.001). Only the multi-ingredient formulation significantly suppressed α-MSH levels (p < 0.001). Regarding melanin reduction, the multi-ingredient formulation achieved comparable efficacy to 4% HQ but outperformed 10% vitamin C (p < 0.05).

Conclusion: The multi-ingredient formulation demonstrated superior efficacy in reducing UVB-induced AA and α-MSH levels while achieving comparable melanin reduction to 4% HQ. These results are consistent with the prevailing consensus that effective melasma management necessitates a multifaceted approach that concurrently targets multiple mechanisms.

## Introduction

Melasma is a chronic, acquired hyperpigmentary disorder that is exacerbated by sun exposure [1]. It is characterized by the appearance of asymptomatic hyperpigmented macules and patches with irregular borders [2]. While melasma can occur in all skin types, it disproportionately affects individuals with darker skin tones, particularly women, and has a significant negative impact on quality of life [3]. The pathogenesis of melasma involves a complex interplay of genetic predisposition, hormonal influences, and exposure to solar ultraviolet radiation (UVR) [4]. These factors collectively drive key pathological changes, including abnormal activation of melanocytes, accumulation of melanin and melanosomes in the epidermis and dermis, increased mast cell density, solar elastosis, enhanced vascularization, and disruption of the basement membrane [5]. Of the UVR that reaches the Earth’s surface, approximately 95% is ultraviolet A (UVA) (320-400 nm), while 5% is UVB (290-320 nm) [6]. UVB radiation primarily interacts with epidermal keratinocytes, inducing direct DNA damage [7]. One key molecular mechanism in melasma involves UVB-induced activation of p53 in response to DNA damage, which triggers keratinocytes to secrete alpha-melanocyte-stimulating hormone (α-MSH) via activation of the proopiomelanocortin promoter [8]. This underscores the importance of broad-spectrum sunscreens as a cornerstone for preventing melasma in high-risk populations [1]. In addition to sunscreens, various active ingredients can complement melasma management by targeting its underlying mechanisms [9]. Antioxidants and free radical scavengers, such as vitamin C, help reduce oxidative stress and mitigate some of the harmful effects of UVR [10]. Depigmenting agents also play an important role in improving the cosmetic appearance of melasma [11]. For instance, niacinamide exhibits antioxidant properties and has shown comparable efficacy to hydroquinone (HQ) - a widely used but safety-limited anti-melanogenic agent [12] - in reducing skin pigmentation when applied topically [13]. Tyrosinase inhibitors such as arbutin can also effectively suppress melanogenesis by reducing the expression and activity of the rate-limiting enzyme in melanin synthesis [14]. Additionally, tranexamic acid (TA), a traditional hemostatic agent, has demonstrated hypopigmentary effects when applied topically to melasma lesions [15]. TA also helps prevent UVR-induced pigmentation by potentially reducing the release of arachidonic acid (AA) [16] and α-MSH [17]. Despite advancements in understanding its pathophysiology, treating melasma remains a significant challenge [1]. In this context, topical formulations that combine multiple active ingredients with complementary mechanisms of action offer promising potential for improved outcomes [18].

Based on this rationale, we designed an in vitro study to evaluate the efficacy of a multi-ingredient blend formulation containing 3% TA, 2% ascorbyl glucoside (AG), 2.5% arbutin, and 5% niacinamide compared to a simple 10% vitamin C preparation in suppressing UVB-induced expression of AA, α-MSH, and melanin in a keratinocyte-melanocyte co-culture model. A 4% HQ preparation was included as a positive control in all experiments to benchmark the efficacy of these formulations [13].

## Materials and methods

Materials

The skin-lightening blend formulation (Agex Serum Spot; Bionativa SpA, Barberino Tavarnelle, Italy) and the topical 10% vitamin C preparation (Vitamin C Brightening Night Serum; Garnier SkinActive, Paris, France) were obtained from commercial retailers. The 4% HQ preparation was freshly prepared by a local pharmacy. Molecular biology-grade distilled water (DW) (Sigma, St. Louis, USA) was employed as the negative control.

Experimental protocol

The keratinocyte-melanocyte co-culture was established following a previously described protocol [19]. Briefly, normal human epidermal melanocytes (CellSystems, Troisdorf, Germany) and keratinocytes (PromoCell, Heidelberg, Germany) were used between passages two and five and maintained in DermaLife Basal Medium (CellSystems, Troisdorf, Germany) supplemented with DermaLife M Medium (Lifeline Cell Technology, Frederick, USA) for melanocytes or DermaLife K Medium (Lifeline Cell Technology, Frederick, USA) for keratinocytes. For co-culture preparation, keratinocytes and melanocytes were harvested separately using an identical trypsinization method. The cells were seeded onto culture dishes at a physiological keratinocyte-to-melanocyte ratio of 10:1 [20] to replicate the epidermal microenvironment and incubated at 37°C with 5% CO_2_ for 24 h. Twenty-four hours prior to UVB irradiation, the medium was replaced with fresh medium containing one of the following test solutions at a concentration of 2% v/v: (i) the blend formulation, (ii) the 10% vitamin C preparation, (iii) the 4% HQ preparation, or (iv) DW (negative control). The cells were then exposed to UVB radiation at a dose of 150 mJ/cm^2^ for 10 min using a Spectrolinker XL-1500 UV crosslinker (Spectronics, Westbury, USA), which emits primarily in the UVB range (280-320 nm; peak: 312 nm). After irradiation, the cells were incubated in a fresh medium until further analysis. Unirradiated cells served as reference controls.

Quantification of AA, α-MSH, and melanin content in co-culture cell lysates

At 48 h after UVB irradiation, keratinocyte-melanocyte co-culture samples - containing approximately 3 × 10^6 ^cells - underwent lysis in an appropriate buffer (200 μL), followed by a 10 min centrifugation at 13,000 × g. We subsequently measured AA (Abcam Ltd., Cambridge, UK) and α-MSH (Abbexa Ltd., Cambridge, UK) concentrations in cell lysates using commercially available enzyme-linked immunosorbent assay (ELISA) kits according to the manufacturer’s protocol. Melanin content was quantified using a fluorometric assay kit (Sigma, St. Louis, USA).

Statistical analysis

Following an established methodology [7], biomarker measurements were expressed in arbitrary units (a.u.), with unirradiated control cells serving as the baseline, standardized to a value of one. Continuous data are expressed as mean ± standard deviation (SD) from at least three independent experiments conducted in duplicate. Statistical comparisons between experimental groups were performed using one-way analysis of variance (ANOVA), followed by pairwise Tukey’s post hoc tests. All calculations were conducted using IBM SPSS Statistics version 20.0 (IBM, Armonk, USA), with two-tailed p-values < 0.05 considered statistically significant.

## Results

Analysis of UVB-induced changes in AA, α-MSH, and melanin content

Following UVB irradiation, the levels of AA, α-MSH, and melanin were significantly increased in cell lysates from the keratinocyte-melanocyte co-culture treated with DW (negative control) compared to unirradiated control cells (Table 1). Specifically, AA, α-MSH, and melanin levels increased by 4.3, 3.7, and 2.6-fold, respectively (all p < 0.001). To evaluate whether pretreatment with the blend formulation, 10% vitamin C, or 4% HQ could mitigate UVB-induced changes in the three biomarkers, their levels were measured in co-cultured cells pretreated with each preparation prior to UVB exposure.

**Table 1:** Arachidonic acid, α-MSH, and melanin content in cells from the keratinocyte-melanocyte co-culture exposed to different experimental conditions Data are expressed as means ± standard deviations. The negative control cells were conventionally set at one. In all analyses, two-tailed p-values < 0.05 were considered statistically significant. *p < 0.001 versus distilled water (Tukey's post hoc test); †p < 0.001 versus 10% vitamin C (Tukey's post hoc test) and 4% hydroquinone (Tukey's post hoc test); ‡p < 0.05 versus distilled water (Tukey's post hoc test); §p < 0.05 versus 10% vitamin C (Tukey's post hoc test). UVB, ultraviolet B; α-MSH, alpha-melanocyte-stimulating hormone; ANOVA, analysis of variance; a.u., arbitrary units

Experimental Condition	UVB Irradiation	Arachidonic Acid (a.u.)	α-MSH (a.u.)	Melanin (a.u.)	F-value (ANOVA)	p-value (ANOVA)
Negative Control	No	1.0	1.0	1.0	-	-
Distilled Water	Yes	4.3 ± 0.7	3.7 ± 0.3	2.6 ± 0.5	42.8	p < 0.001
Blend Formulation	Yes	2.3 ± 0.4*,†	2.5 ± 0.5*	1.8 ± 0.3*,§	56.2	p < 0.001
10% Vitamin C	Yes	3.4 ± 0.9*	3.6 ± 0.4	2.2 ± 0.5‡	38.7	p < 0.001
4% Hydroquinone	Yes	3.2 ± 0.8*	3.4 ± 0.6	1.6 ± 0.4*,§	47.5	p < 0.001

AA levels

**Figure 1:**
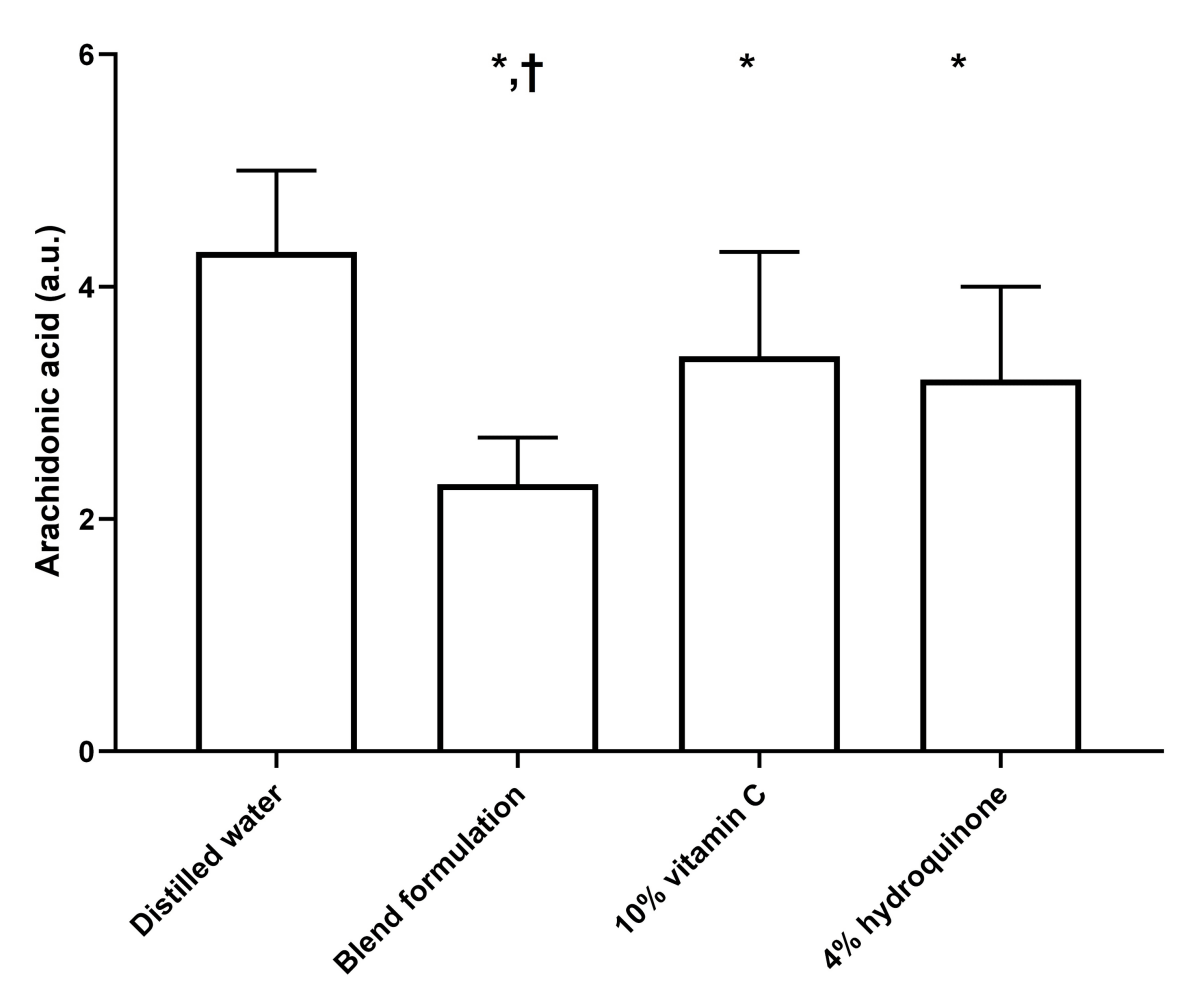
Effect of distilled water, blend formulation, 10% vitamin C, and 4% hydroquinone on UVB-induced arachidonic acid formation in cells from the keratinocyte-melanocyte co-culture The bar graph represents arachidonic acid levels under different conditions. Bars indicate mean values, with error bars representing standard deviations. In all analyses, two-tailed p values < 0.05 were considered statistically significant. *p < 0.001 compared to distilled water (Tukey's post hoc test); †p < 0.001 compared to 10% vitamin C (Tukey's post hoc test) and 4% hydroquinone (Tukey's post hoc test). a.u., arbitrary units; UVB, ultraviolet B

Figure 1 illustrates the protective effects of the blend formulation, 10% vitamin C, and 4% HQ in mitigating the UVB-induced increase in AA levels in cells from the keratinocyte-melanocyte co-culture. The ANOVA test revealed a significant pretreatment effect (p < 0.001). Post hoc analyses demonstrated that all tested preparations significantly reduced AA levels compared to the distilled water-treated negative control (all p < 0.001). However, the blend formulation was significantly more effective than both 10% vitamin C and 4% HQ in reducing AA levels (p < 0.001).

α-MSH levels

**Figure 2:**
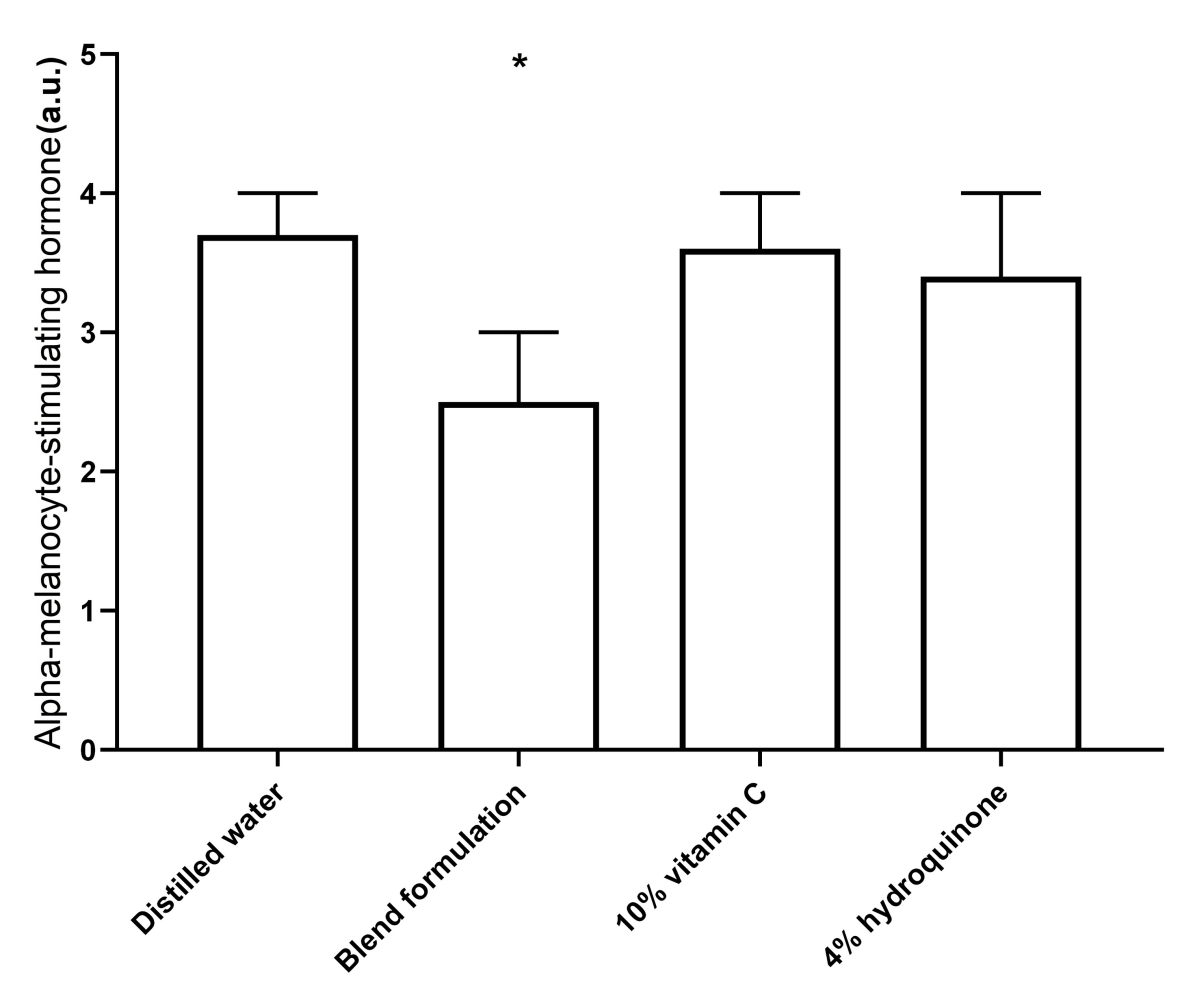
Effect of distilled water, blend formulation, 10% vitamin C, and 4% hydroquinone on UVB-induced α-MSH formation in cells from the keratinocyte-melanocyte co-culture The bar graph represents alpha-melanocyte-stimulating hormone levels under different conditions. Bars indicate mean values, with error bars representing standard deviations. In all analyses, two-tailed p values < 0.05 were considered statistically significant. *p < 0.001 compared to distilled water (Tukey's post hoc test). a.u., arbitrary units; UVB, ultraviolet B; α-MSH, alpha-melanocyte-stimulating hormone

The effects of the blend formulation, 10% vitamin C, and 4% HQ on α-MSH levels in UVB-irradiated cells from the keratinocyte-melanocyte co-culture are depicted in Figure 2. While the ANOVA test indicated a significant pretreatment effect (p < 0.001), Tukey’s post hoc tests revealed that only the blend formulation showed a statistically significant reduction compared to distilled water-treated cells (p < 0.001). Neither 10% vitamin C nor 4% HQ significantly altered α-MSH content in cell lysates.

Melanin content

**Figure 3:**
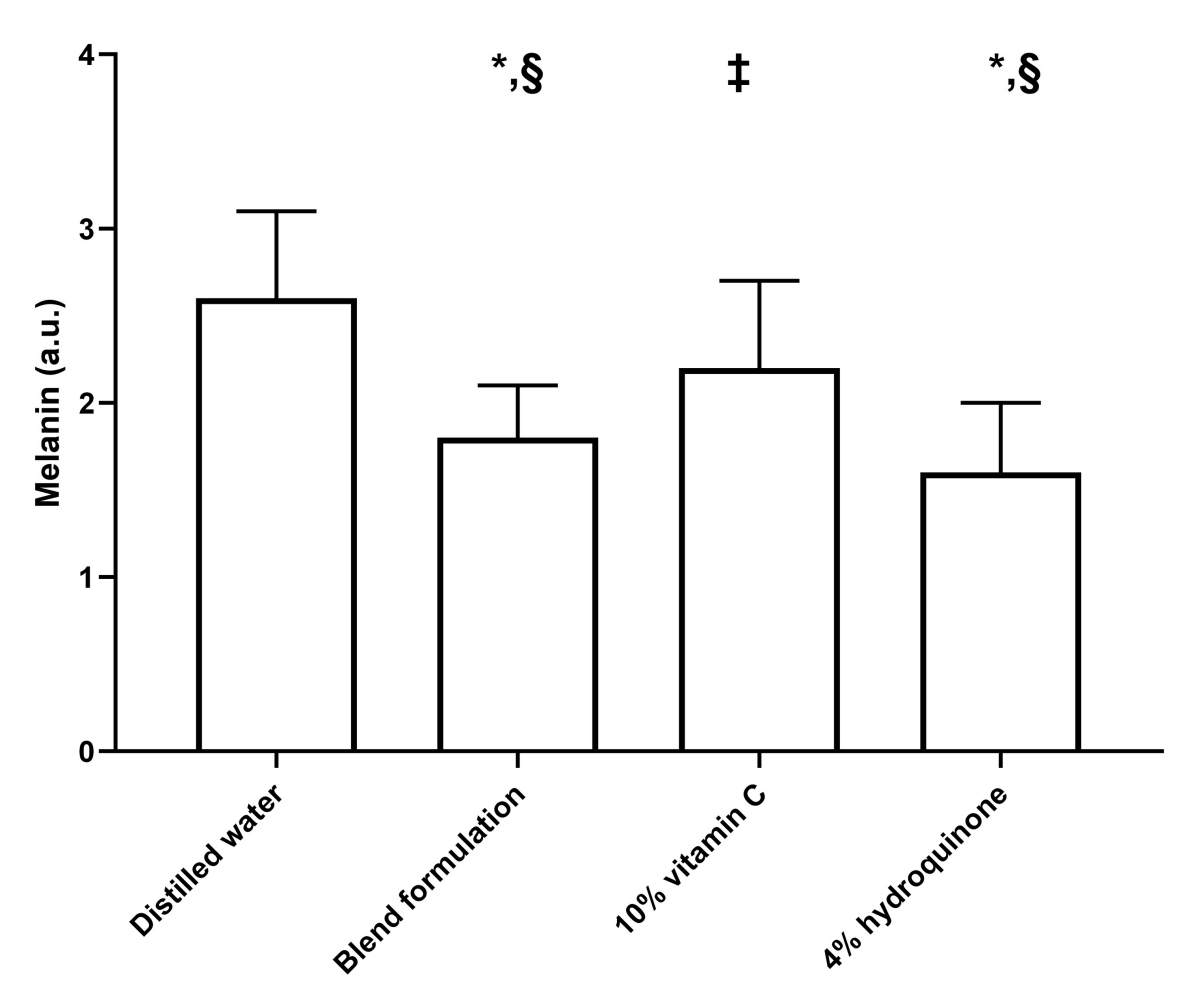
Effect of distilled water, blend formulation, 10% vitamin C, and 4% hydroquinone on UVB-induced melanin formation in cells from the keratinocyte-melanocyte co-culture The bar graph represents melanin levels under different conditions. Bars indicate mean values, with error bars representing standard deviations. In all analyses, two-tailed p values < 0.05 were considered statistically significant. *p < 0.001 compared to distilled water (Tukey's post hoc test); ‡p < 0.05 compared to distilled water (Tukey's post hoc test); §p < 0.05 compared to 10% vitamin C (Tukey's post hoc test). a.u., arbitrary units; UVB, ultraviolet B

Figure 3 presents the effects of the tested formulations on melanin content in cells derived from the keratinocyte-melanocyte co-culture. A significant pretreatment effect was evident on ANOVA (p < 0.001), and Tukey's post hoc tests revealed that all compounds significantly reduced melanin content compared to DW-treated cells. However, the extent of reduction varied significantly across the tested preparations. Specifically, the blend formulation and 4% HQ were significantly more effective than 10% vitamin C in lowering melanin content (both p < 0.05). There were no significant differences in terms of melanin reduction between 4% HQ and the blend formulation.

## Discussion

Given the chronic and refractory nature of melasma, successful management frequently necessitates targeting multiple pathways simultaneously to achieve satisfactory clinical outcomes [21]. This in vitro study utilized a straightforward keratinocyte-melanocyte co-culture model with UVB irradiation to compare the effects of a multi-ingredient blend (3% TA, 2% AG, 2.5% arbutin, and 5% niacinamide) against two simpler formulations (10% vitamin C and 4% HQ) on three biomarkers of interest. Our investigation yielded several key findings. First, while all tested preparations significantly reduced UVB-induced AA formation in the keratinocyte-melanocyte co-culture cells compared to the negative control, the multi-ingredient preparation demonstrated superior efficacy relative to both comparators. Second, the blend uniquely decreased α-MSH levels in keratinocyte-melanocyte lysates, whereas neither 10% vitamin C nor 4% HQ exhibited this effect. Third, regarding melanin content reduction, the multi-ingredient blend formulation significantly outperformed 10% vitamin C while achieving comparable efficacy to the benchmark 4% HQ preparation. Collectively, these results indicate that a preparation containing multiple skin-lightening ingredients has the potential to achieve optimal anti-melanogenic effects through complementary mechanisms of action.

AA and its metabolites play a fundamental role in melanocyte activation, orchestrating both melanin synthesis and cellular proliferation in response to UVR exposure [22]. These signaling cascades are integral to the pathogenesis of both melasma and post-inflammatory hyperpigmentation [9]. In our experimental model, UVB irradiation of keratinocyte-melanocyte co-cultures induced a 4.3-fold increase in AA expression compared to unirradiated controls. When the keratinocyte-melanocyte co-culture was pretreated with either 10% vitamin C or 4% HQ, AA levels in cell lysates decreased significantly by 20.9% and 25.6%, respectively. These observations are consistent with prior evidence that both agents are capable of inhibiting AA release [23,24]. However, the blend formulation demonstrated superior efficacy, achieving a 46.5% reduction in AA levels. This notable effectiveness is likely attributable to the complementary mechanisms of AG and TA. While AG, a stable vitamin C derivative, exerts direct antioxidant and anti-inflammatory effects [25], TA distinctly reduces AA synthesis via inhibition of the plasminogen/plasmin cascade [26]. Additionally, by interfering with plasminogen activity and modulating keratinocyte-melanocyte signaling, TA can uniquely suppress α-MSH production from irradiated keratinocytes [17,27]. This specific property explains why only the TA-containing formulation effectively counteracted the UVB-induced elevation in α-MSH levels, whereas both 10% vitamin C and 4% HQ pretreatments showed no significant impact on this parameter. Regarding melanin production following UVB exposure, the blend formulation achieved an 18.2% reduction compared to unirradiated controls. Although numerically lower than the 27.3% reduction observed with 4% HQ - which represents the first-line topical treatment for melasma [28] - this difference did not reach statistical significance. Notably, HQ is believed to exert its skin-lightening effects through multiple mechanisms, including inhibiting the activity of tyrosinase, altering melanosome formation, and increasing their degradation, as well as inhibiting DNA and RNA synthesis [29]. In addition to decreasing AA and α-MSH release, it is likely that the mechanisms by which the multi-ingredient blend might reduce melanin synthesis following UVB irradiation can also involve tyrosinase activity inhibition - mediated by arbutin [14] - and inhibition of melanosome transfer from melanocytes to keratinocytes - elicited by niacinamide [30]. This multifaceted approach underscores the importance of targeting diverse pathways in melanogenesis to achieve optimal therapeutic outcomes. Collectively, our data highlight the potential advantages of complex formulations containing multiple active ingredients with complementary mechanisms of action. The synergistic effects of TA, AG, arbutin, and niacinamide not only achieved comparable melanin reduction to the reference-standard 4% HQ but also uniquely decreased α-MSH levels and demonstrated enhanced suppression of UVB-induced AA formation. These results are consistent with the prevailing consensus that effective melasma management necessitates a multifaceted therapeutic strategy that concurrently targets multiple pathogenic mechanisms [2,18].

Several limitations of this study should be acknowledged. First, the in vitro keratinocyte-melanocyte co-culture model, while useful for mechanistic investigations, cannot fully replicate the complex interactions and microenvironment present in human skin. Accordingly, the model lacks important structural elements like the dermal-epidermal junction, blood vessels, and immune cells that play crucial roles in melasma pathogenesis [4]. In particular, the vascular component of melasma, which has been increasingly recognized as a significant contributor to its pathogenesis, was not addressed in this study. Second, the acute UVB exposure protocol used in our investigation may not accurately reflect the chronic, cumulative UV damage typically associated with melasma development in clinical settings. Third, the study evaluated only short-term responses, whereas melasma is a chronic condition that necessitates long-term management. Fourth, the concentrations of active ingredients used in the formulations, while based on commercially available products, may achieve different tissue levels when applied topically to human skin due to variations in penetration and bioavailability. Finally, while the co-culture model demonstrated promising results for the multi-ingredient formulation, clinical efficacy cannot be directly extrapolated from these findings without subsequent in vivo validation through properly designed trials. Future studies would also benefit from conducting comparative analyses with other widely used treatments for melasma, such as niacinamide and kojic acid, evaluated as standalone ingredients. Notably, the question of whether the tested formulations may be effective in addressing deep or dermal melasma remains unresolved. Accordingly, this condition is often more resistant to treatment and typically necessitates targeted approaches, such as dermal-specific therapies like microneedling radiofrequency or other advanced modalities.

## Conclusions

This in vitro study demonstrates that a multi-ingredient formulation targeting diverse pathways in melanogenesis shows promising efficacy, achieving comparable melanin reduction to 4% HQ while offering distinct advantages in modulating inflammatory mediators like AA and α-MSH. While these results are encouraging, further in vivo validation is essential to establish the clinical relevance of the formulation tested in our investigation, particularly given melasma's notorious tendency for recurrence and therapeutic resistance. Our findings may provide a rational framework for developing topical treatments that exploit the synergistic effects of multiple active ingredients to improve clinical outcomes in melasma management.
